# Symptoms related to gastrointestinal tract involvement and low muscularity in systemic sclerosis

**DOI:** 10.1007/s10067-022-06059-5

**Published:** 2022-02-11

**Authors:** Edoardo Rosato, Antonietta Gigante, Chiara Pellicano, Annalisa Villa, Francesco Iannazzo, Danilo Alunni Fegatelli, Maurizio Muscaritoli

**Affiliations:** 1 Department of Translational and Precision Medicine, “Sapienza” University of Rome, Viale dell’ Università 37, 00185 Rome, Italy; 2 Department of Public Health and Infectious Diseases, “Sapienza” University of Rome, Rome, Italy

**Keywords:** Fat-free mass, Gastrointestinal, Microbiota, Sarcopenia, Systemic sclerosis

## Abstract

**Introduction/objectives:**

Gastrointestinal tract (GIT) involvement is frequently observed in systemic sclerosis (SSc) and may lead to nutritional impairment. The aim of the study was to assess the prevalence of symptoms related to GIT involvement and to analyze the possible association between gastrointestinal symptoms and low muscularity in SSc patients.

**Methods:**

Sixty-nine consecutive patients (60 females, median age 53 (IQR 43–63), body mass index (BMI) 23.2 (IQR 20.9–24.6) kg/m^2^) with diagnosis of SSc admitted to our Scleroderma Unit were enrolled. Clinical status, anthropometric data, and bioelectrical impedance (Inbody 770, USA) analysis-assessed Fat-Free Mass Index (FFMI) were recorded upon enrollment. UCLA questionnaire was used to quantify GIT involvement with seven specific scales.

**Results:**

Mean FFMI was 16.2 kg/m^2^ (IQR 15.2–17.6). The median UCLA total score was 0.53 (IQR 0.19–0.89). FFMI showed a significant negative correlation with UCLA total score (*r* = −0.29, *p* = 0.016) and UCLA distention/bloating (*r* = −0.35, *p* < 0.01). In 16 patients (23.1%), FFMI was reduced and UCLA distention/bloating was significantly higher (*p* = 0.039) in SSc patients with lower FFMI [1.75 (IQR 0.75–2.12) vs 0.75 (IQR 0.25–1.75)]. At multiple linear regression model, FFMI showed association with UCLA distention/bloating [beta coefficient − 0.315 (95% CI of beta coefficient: −0.591; −0.039), *p* = 0.026], BMI [beta coefficient 0.259 (95% CI of beta coefficient: 0.163; 0.355), *p* = 0.001], and disease duration [beta coefficient − 0.033 (95% CI of beta coefficient: −0.059; −0.007), *p* = 0.015].

**Conclusions:**

In SSc, low FFMI is associated with symptoms related to GIT involvement, in particular with distension/bloating.

**Table:** 

**Key Points** *• FFMI is associated with symptoms related to GIT involvement.* *• Low FFMI is associated with symptoms related to UCLA distention/bloating.* *• Malnutrition is not associated with symptoms related to GIT involvement.*

## Introduction

Systemic sclerosis (SSc) is a rare autoimmune disease characterized by microvascular damage, production of autoantibodies, and fibrosis in the skin and internal organs, which is highly prevalent in female subjects, with a female:male ratio of approximately 10:1. Visceral involvement impinges quality of life and negatively impacts on prognosis of SSc patients. Interstitial lung disease, pulmonary arterial hypertension, scleroderma renal crisis, and gastrointestinal tract (GIT) involvement represent some of the most typical manifestations of the disease [1]. Any part of the GIT can be involved and approximately 90% of SSc patients present gastrointestinal manifestations. Gastrointestinal assessment includes the lactulose hydrogen breath test to evaluate small intestine motility and detection of small intestinal bacterial overgrowth (SIBO), endoscopic or motility capsules to evaluate small intestine transit time. Other investigations to study GIT include X-rays, barium swallow test, and manometry [2]. The presence of gastrointestinal symptoms and their impact on quality of life of scleroderma patients are assessed by validated questionnaires such as the University of California, Los Angeles, Scleroderma Clinical Trials Consortium Gastrointestinal Scale (UCLA SCTC GIT 2.0) [3].

Early manifestations of GIT involvement encompass a number of motility and transit disorders which affect both upper and lower GIT. Dysmotility, SIBO, and malabsorption are believed to play a role in the pathogenesis of altered nutritional status observed in SSc patients [4]. Among malnutrition-related abnormalities, low muscle mass is common in SSc patients, with a prevalence of up to 22% [5] when diagnosed as having low lean mass and low muscle strength [5], according to the European Working Group on Sarcopenia in Older People criteria [6, 7].

If muscle function is not measured, low muscle mass, as it may be assessed by imaging techniques or estimated by bioimpedance analysis (BIA), should be better referred to as low muscularity [8].

Although there is increasing interest in this topic, the role of GIT involvement in the pathogenesis of body composition changes (e.g., low muscularity) in SSc is not entirely clear.

The aim of the study was to assess the prevalence of symptoms related to GIT involvement and to analyze the possible association between gastrointestinal symptoms and low muscularity in SSc patients.

## Materials and methods

### Study population

One hundred and two SSc patients admitted to Scleroderma Unit of Department of Translational and Precision Medicine and classified according to American College of Rheumatology/European League Against Rheumatism criteria [9] were considered.

Out of the 102 SSc patients, 33 were excluded from this study: 26 did not sign informed consent, 2 had myositis, 1 had increased serum CPK levels, 1 had eating disorders, and 3 had a previous diagnosis of neoplastic disease. Therefore, a total of 69 SSc patients were included in the present analysis.

Clinical assessment, biochemical analyses, anthropometric data, body composition, and symptoms related to GIT involvement were recorded in all patients at the time of enrollment.

Both the inflammatory indices erythrocyte sedimentation rate (ESR) and C-reactive protein (CRP) were recorded. Reference values were 0–20 mm/h and 100–6000 mcg/L for ESR and CRP, respectively.

Exclusion criteria were as follows: patients aged <18 or > 70 years, previous or current diagnosis of malignant disease, and eating disorders.

All SSc patients were undergoing treatment with calcium channel blockers (nifedipine 30 mg/day). None of the patients was treated with immunosuppressive agents in the previous 6 months.

The patients on steroid treatment included in the present investigation were receiving minimal doses of nonfluorinated glucocorticoid (<10 mg/day) preparations (in the previous 6 months) that are rarely associated with glucocorticoid-induced myopathy [10].

The subjects’ written consent was obtained according to the Declaration of Helsinki and the study was conducted in agreement to local ethics committee’s directives.

#### Clinical assessment

The following data have been collected: limited cutaneous (lc)SSc and diffuse cutaneous (dc)SSc form [11], modified Rodnan skin score (mRss) for the skin thickening [12], disease severity scale (DSS) [13], disease activity index (DAI) [14], and history of digital ulcers (DUs) [15]. Nailfold videocapillaroscopy was performed with videocapillaroscope (software Pinnacle Studio Version 8) equipped with a 500× optical probe. The patterns identified within the “SSc pattern” include early, active, and late [16].

#### Anthropometric data

Body mass index (BMI, weight/height) was calculated from weight (kg) and height (m) and expressed in kg/m^2^.

#### Bioelectrical impedance analysis

Bioelectrical impedance analysis was performed in all SSc patients using a research-grade multifrequency body composition analyzer applying an alternating electric current of 800 μA at 50-Hz frequency (Inbody 770, USA). Blinded physicians performed BIA in the morning in fasting condition and Fat-Free Mass Index (FFMI, expressed in kg/m^2^) was calculated using a program provided by the producer. The sex-specific FFMI cut-off values for reduced muscularity were as follows: <15 kg/m^2^ (females) and < 17 kg/m^2^ (males) [17].

#### Nutritional assessment

The risk of malnutrition, evaluated with Malnutrition Universal Screening Tool (MUST), includes BMI (kg/m^2^) (BMI > 20 = 0; BMI 18.5–20 = 1; BMI < 18.5 = 2), unplanned weight loss in the previous 3 months, and the presence of acute disease or no food intake for >5 days. The sum of the scores from each category for the risk of malnutrition is as follows: 0 = low, 1 = medium, and 2 = high [18].

The diagnosis of malnutrition was evaluated using both European Society of Clinical Nutrition and Metabolism (ESPEN) [17] and Global Leadership Initiative on Malnutrition (GLIM) criteria [19].

##### ESPEN

In the assessment of malnutrition, ESPEN criteria include a BMI < 18.5 or unintentional weight loss >10% indefinite time or > 5% in the previous 3 months combined with a BMI <20 if 70 years of age or < 22 if ≥70 years of age or FFMI (kg/m^2^) < 15 and 17 in women and men, respectively [17].

##### Glim

In the assessment of malnutrition, GLIM criteria include require ≥1 phenotypic and ≥ 1 etiologic criterion. Phenotypic criteria include unintentional weight loss >5% within the past 6 months or > 10% beyond 6 months; low BMI, <20 if <70 years or < 22 if >70 years; low FFMI, <15 if female and < 17 if male. Etiologic criteria include disease burden/inflammatory condition and reduced food intake or assimilation [19]. Grading of malnutrition, stage 1/moderate and 2/severe, was based on either BMI values or % weight loss, according to GLIM criteria [19].

#### UCLA SCTC GIT 2.0

UCLA SCTC GIT 2.0 questionnaire was administered to all patients and contains seven specific scales (reflux, distention/bloating, diarrhea, fecal soilage, constipation, emotional well-being, and social functioning) with 34 items on symptoms of GIT involvement and the impact on quality of life (QOL). The scales are scored from 0.0 (better QOL) to 3.0 (worse QOL). Only diarrhea and constipation scales range from 0.0 to 2.0 and 0.0 to 2.5, respectively. The total GIT score is obtained from average of 6 of 7 scales (except constipation) and it is scored from 0.0 (better QOL) to 3.0 (worse QOL). According to UCLA SCTC GIT 2.0 scales, the values to identify the self-rated severity on symptoms of GIT involvement are as follows: reflux >1.23; distention /bloating >1.75; diarrhea >1.19; fecal soilage >0.77; constipation >0.51; emotional well-being >1.54; social functioning >0.84; and total GIT Score > 1.31 [3].

### Statistical analysis

Descriptive statistics were used to characterize the study population: continuous variables were represented as median (interquartile range, IQR); numbers and percentages were used for describing categorical data. For numerical variables, the Wilcoxon signed rank test was applied to detect significant differences in the two study groups. For categorical data, the chi-squared test was used to compare differences in proportions between two groups. Scatter plots were used to describe the relationship between numerical variables and the strength of the linear relationship was measured using Pearson’s correlation coefficient. Finally, a multiple linear regression model was used to determine the most predictor variables for FFMI (we established the final regression model using a stepwise procedure selecting the best model based on the AIC). A *p* value <0.05 was considered significant. Statistical analysis was performed using the statistical software R (version 4.0.4).

## Results

Sixty-nine patients (9 males, median age 53 (IQR 43–63)) were enrolled in the study: 33 (47.8%) had dcSSc and 36 (52.2%) lcSSc. Table 1 shows the clinical features of the SSc patients studied. The median value of FFMI (kg/m^2^) was 16.2 (IQR 15.2–17.6). The median value of FFMI (kg/m^2^) for women was 15.9 (IQR 15–17) while for men was 18.9 (IQR 17.8–20.4). In 16 patients (23.2%), FFMI were below the cut-off values for the diagnosis of low muscle mass. In 15 of 60 women (25%) and in 1 of 9 men (11.1%), FFMI were below the cut-off values for the diagnosis of low muscularity. This difference, however, was not statistically significant (*p* = 0.357 at Pearson’s chi-square test).

**Table 1:** Clinical characterization of the study population

Age in years, median (IQR)	53 (43–63)
Female gender, *n* (%)	60 (87)
Disease duration in years, median (IQR)	10 (6–14)
Cutaneous form	
dcSSc, *n* (%)	33 (47.8)
lcSSc, *n* (%)	36 (52.2)
mRss, median (IQR)	10 (6-18)
DAI, median (IQR)	1.5 (0.9-3.2)
DSS, median (IQR)	6 (4-6)
NVC	
Early, *n* (%)	16 (23.2)
Active, *n* (%)	24 (34.8)
Late, *n* (%)	29 (42)
Autoantibodies	
Anti-centromere, *n* (%)	27 (39.1)
Anti-topoisomerase I, *n* (%)	33 (47.8)
No specific autoantibodies, *n* (%)	9 (13.1)
ESR in mm/h, median (IQR)	25 (13–37)
ESR > 20 mm/h, *n* (%)	43 (62.3)
CRP in mcg/L, median (IQR)	1600 (900–3000)
CRP > 6000 mcg/L, *n* (%)	10 (14.5)
Dlco/Va, median (IQR)	84 (72–92)
sPAP, median (IQR)	28 (25–32)
PAH, *n* (%)	5 (7.2)
BMI (kg/m^2^), median (IQR)	23.2 (20.9–24.6)
Malnutrition ESPEN, *n* (%)	8 (11.6)
Malnutrition GLIM, *n* (%)	16 (23.2)
Digital ulcers history, *n* (%)	33 (47.8)
Digital pitting scars, *n* (%)	28 (40.6)
Raynaud’s requiring vasodilatators therapy, *n* (%)	69 (100)
PPI therapy, *n* (%)	69 (100)
Prokinetic therapy, *n* (%)	14 (20.3)
Steroid therapy, *n* (%)	27 (39.1)

*dcSSc*, diffuse cutaneous systemic sclerosis; *lcSSc*, limited cutaneous systemic sclerosis; *mRss*, modify Rodnan Skin Score; *DAI*, disease activity index; *DSS*, disease severity index; *NVC*, nailfold videocapillaroscopy; *ESR*, erythrocyte sedimentation rate; *CRP*, c-reactive protein; *Dlco/Va*, diffusing capacity of the lungs for carbon monoxide divided by the alveolar volume; *sPAP*, systolic pulmonary arterial pressure; *PAH*, pulmonary arterial hypertension; *BMI*, body mass index; *PPI*, proton pump inhibitors; *IQR*, interquartile range

According to the UCLA SCTC GIT 2.0 questionnaire, 9 of 69 (13%) patients had a total score compatible with severe gastrointestinal symptoms; 20 of 69 (28.9%) had severe reflux; 24 of 69 (34.7%) had severe abdominal distension symptoms; 6 of 69 (8.6%) had severe diarrhea; 15 of 69 (21.7%) had severe fecal soilage; 10 of 69 (14.4%) had severe problems in the emotional well-being; and 13 of 69 (18.8%) severe limitations in social function. Table 2 shows the UCLA SCTC GIT 2.0 total score and single items.

**Table 2:** University of California, Los Angeles, Scleroderma Clinical Trials Consortium Gastrointestinal Scale (UCLA SCTC GIT 2.0) questionnaire values

UCLA SCTC GIT 2.0 total score, median (IQR)	0.53 (0.19–0.89)
UCLA SCTC GIT 2.0 reflux, median (IQR)	0.62 (0.25–1.25)
UCLA SCTC GIT 2.0 distention/bloating, median (IQR)	1 (0.44–1.81)
UCLA SCTC GIT 2.0 fecal soilage, median (IQR)	0 (0–0)
UCLA SCTC GIT 2.0 diarrhea, median (IQR)	0 (0–0.5)
UCLA SCTC GIT 2.0 social functioning, median (IQR)	0.24 (0–0.66)
UCLA SCTC GIT 2.0 emotional well-being, median (IQR)	0.28 (0–0.80)

Regarding the risk of malnutrition, MUST score = 0 was found in 51 patients (73.9%), MUST score = 1 was found in 8 patients (11.6%), and MUST score ≥ 2 was found in 10 of the 69 patients (14.5%).

Malnutrition according to ESPEN criteria was diagnosed in 8 of 69 patients (11.6%), whereas malnutrition according to GLIM criteria was found in 16 of 69 patients (23.2%).

GLIM severity grading of malnutrition was moderate (stage 1) in 11 (68.8%) and severe (stage 2) in 5 (31.2%) of the 16 malnourished patients according to BMI and % of weight loss.

Table 3 shows the comparative analysis of disease variables and UCLA SCTC GIT 2.0 score (total and single items) between SSc patients with low and normal FFMI. No significant differences were found between anti-centromere and anti-topoisomerase-I auto-antibodies and subsets of disease (diffuse and limited) between patients with low and normal FFMI.

**Table 3:** Comparative analysis between SSc patients with low and normal FFMI

Variable	Low FFMI*, *n* = 16	Normal FFMI, *n* = 53	*p*
Age in years, median (IQR)	51 (42–57)	54 (43–65)	0.594
Female gender, *n* (%)	15/16 (93.8)	45/53 (84.9)	0.619
Disease duration in years, median (IQR)	12 (10–25)	9 (6–13)	0.02
Cutaneous form			
dcSSc, *n* (%)	11/16 (68.7)	22/53 (41.5)	0.086
lcSSc, *n* (%)	5/16 (31.3)	31/53 (58.5)	0.086
mRss, median (IQR)	16 (9–20)	9 (6–16)	0.046
DAI, median (IQR)	3.1 (1.5–5.4)	1.5 (0.8–2.5)	0.013
DSS, median (IQR)	6 (5–7)	5 (4–6)	0.047
NVC			
Early, *n* (%)	3/16 (18.8)	13/53 (24.5)	0.150
Active, *n* (%)	3/16 (18.8)	21/53 (39.6)	0.150
Late, *n* (%)	10/16 (62.5)	19/53 (35.8)	0.150
Autoantibodies			
Anti-centromere, *n* (%)	5/16 (7.9)	22/53 (31.9)	0.725
Anti-topoisomerase I, *n* (%)	9/16 (13)	24/53 (34.8)	0.725
No specific autoantibodies, *n* (%)	2/16 (2.9)	7/53 (10.1)	0.725
ESR in mm/h, median (IQR)	25.5 (14–33)	25 (13–38)	0.955
ESR > 20 mm/h, *n* (%)	10/16 (62.5)	33/53 (62.3)	1
CRP in mcg/L, median (IQR)	1700 (1175–2600)	1500 (900–3300)	0.966
CRP > 6000 mcg/L, *n* (%)	2/16 (12.5)	8/53 (15.1)	1
Dlco/Va, median (IQR)	81 (70.5–88.5)	84 (72–95)	0.499
sPAP, median (IQR)	28 (27–32)	28 (25–32)	0.420
PAH, *n* (%)	1/16 (6.3)	4/53 (7.5)	1
BMI (kg/m^2^), median (IQR)	19.0 (18.4–20.0)	23.7 (22.7–24.9)	<0.001
Malnutrition ESPEN, *n* (%)	7 (10.1)	1 (1.9)	<0.001
Malnutrition GLIM, *n* (%)	13 (18.8)	3 (4.3)	<0.001
Digital ulcers history, *n* (%)	10/16 (62.5)	19/53 (35.8)	0.109
Digital pitting scars, *n* (%)	8/16 (50)	20/53 (37.7)	0.558
Raynaud’s requiring vasodilatators therapy, *n* (%)	16/16 (100)	53/53 (100)	N.A.
PPI therapy, *n* (%)	16/16 (100)	53/53 (100)	N.A.
Prokinetic therapy, *n* (%)	4/16 (25)	10/53 (18.9)	0.857
Steroid therapy, *n* (%)	6/16 (37.5)	21/53 (39.6)	1.000
UCLA SCTC GIT 2.0 total score, median (IQR)	0.54 (0.41–0.69)	0.48 (0.19–0.96)	0.490
UCLA SCTC GIT 2.0 reflux, median (IQR)	0.62 (0.25–1.06)	0.62 (0.25–1.25)	0.923
UCLA SCTC GIT 2.0 distention/bloating, median (IQR)	1.75 (0.75–2.12)	0.75 (0.25–1.75)	0.039
UCLA SCTC GIT 2.0 fecal soilage, median (IQR)	0	0	–
UCLA SCTC GIT 2.0 diarrhea, median (IQR)	0 (0.0–0.5)	0 (0.0–0.5)	0.289
UCLA SCTC GIT 2.0 social functioning, median (IQR)	0.16 (0–0.66)	0.33 (0.3–0.66)	0.915
UCLA SCTC GIT 2.0 emotional well-being, median (IQR)	0.22 (0.11–0.44)	0.44 (0.0–0.88)	0.892

*dcSSc*, diffuse cutaneous systemic sclerosis; *lcSSc*, limited cutaneous systemic sclerosis; *mRss*, modified Rodnan Skin Score; *DAI*, disease activity index; *DSS*, disease severity index; *NVC*, nailfold videocapillaroscopy; *ESR*, erythrocyte sedimentation rate; *CRP*, c-reactive protein; *Dlco/Va*, diffusing capacity of the lungs for carbon monoxide divided by the alveolar volume; *sPAP*, systolic pulmonary arterial pressure; *PAH*, pulmonary arterial hypertension; *BMI*, body mass index; *PPI*, proton pump inhibitors; *UCLA*, University of California Los Angeles Scleroderma Clinical Trials Consortium Gastrointestinal Scale questionnaire; *IQR*, interquartile range; *N.A.*, not applicable

*Low Fat-Free Mass Index (FFMI) = <15 kg/m^2^ (females) and < 17 kg/m^2^ (males)

(Cederholm T, Bosaeus I, Barazzoni R, Bauer J, Van Gossum A, Kleket S, al. Diagnostic criteria for malnutrition: an ESPEN consensus statement. *Clin Nutr* 2015; 34:335–340)

C-reactive protein and ESR were above the upper limit of normality (ULN) in 10 and 43 out of the 69 patients, respectively. Both these inflammatory indices were not significantly different between the low and normal FFMI groups.

Disease duration, mRss, DAI, and DSS were higher in SSc patients with low FFMI (Table 3).

The UCLA SCTC GIT 2.0 total score did not differ according to FFMI values; conversely, UCLA SCTC GIT 2.0 distention/bloating was significantly higher (*p* = 0.039) in SSc patients with lower FFMI [1.75 (IQR 0.75–2.12) vs 0.75 (IQR 0.25–1.75)].

The UCLA SCTC GIT 2.0 total score did not differ according to ESPEN [0.46 (IQR 0.15–2.33) vs 0.53 (IQR 0.12–1.06), *p* > 0.05], GLIM [0.59 (IQR 0.17–1.81) vs 0.48 (IQR 0.1–1.06), *p* > 0.05], and MUST [0.53 (IQR 0.1–1.39) vs 0.51 (IQR 0.17–0.72) vs 0.34 (IQR 0.06–2.33), *p* > 0.05] criteria for malnutrition and risk for malnutrition.

No significant difference was found between UCLA SCTC GIT 2.0 distention/bloating and risk of malnutrition in SSc patients according to MUST criteria [1 (IQR 0.25–2.75) vs 1 (IQR 0.25–3) vs 1 (IQR 0.50–3), *p* > 0.05].

The UCLA SCTC GIT 2.0 distention/bloating score was higher, although not significantly, in malnourished vs non-malnourished SSc patients, according to both GLIM [1 (IQR 0.50–3) vs 0.75 (IQR 0.25–2) *p* > 0.05] and ESPEN criteria [1 (IQR 0–3) vs 1 (IQR 0.25–2) *p* > 0.05]. Furthermore, SSc patients with stage 2/severe malnutrition according to GLIM criteria showed higher, although not significantly, score of UCLA SCTC GIT 2.0 distention/bloating with respect to SSc patients with stage 1/moderate GLIM criteria [1.63 (0.50–3) vs 1.13 (1–1.75), *p* > 0.05].

**Fig. 1:**
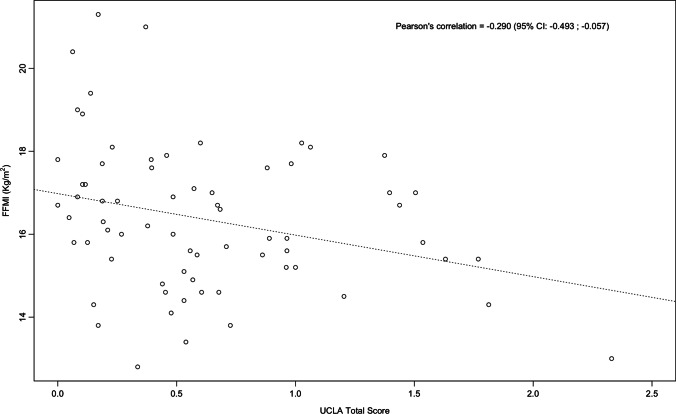
Linear correlation between FFMI and UCLA total score in the 69 patients included in the study. The lines indicate the correlation line and the 95% CI

**Fig. 2:**
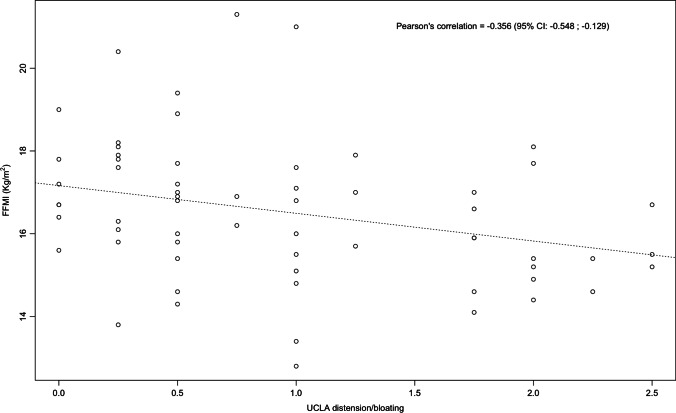
Linear correlation between FFMI and UCLA distention bloating in the 69 patients included in the study. The lines indicate the correlation line and the 95% CI

FFMI showed a significant, albeit weak, negative correlation with UCLA SCTC GIT 2.0 total score (*r* = −0.29, *p* = 0.016) (Fig. 1) and UCLA SCTC GIT 2.0 distention/bloating (*r* = −0.356, *p* = 0.01) (Fig. 2). No significant correlation was observed between FFMI and others single items of UCLA SCTC GIT 2.0. No significant correlation was observed between FFMI and age (*r* = 0.18, *p* > 0.05) or disease duration (*r* = −0.23, *p* > 0.05). A significant, although weak inverse correlation, was observed between FFMI and mRss (*r* = −0.29, *p* = 0.014), DAI (*r* = −0.31, *p* = 0.01), and DSS (*r* = −0.25, *p* = 0.04).

At multiple linear regression model, FFMI showed association with UCLA SCTC GIT 2.0 distention/bloating [beta coefficient − 0.315 (95% CI of beta coefficient: −0.591; −0.039), *p* = 0.026], BMI [beta coefficient 0.259 (95% CI of beta coefficient: 0.163; 0.355), *p* = 0.001], and disease duration [beta coefficient − 0.033 (95% CI of beta coefficient: −0.059; −0.007), *p* = 0.015].

Reduced FFMI was unrelated to steroid use (27 of 69 were receiving minimal steroid doses, <10 mg/day) at multiple linear regression [beta coefficient − 0.424 (95% CI of beta coefficient: −0.885; 0.036), *p* > 0.05].

## Discussion

In the present study, low FFMI was observed in 23% of patients, consistently with literature data reporting a prevalence of low muscularity of 20–23% in SSc patients [5, 20]. In SSc patients with low muscularity, we observed higher scores of skin thickening, disease severity, and activity with respect to non-muscle-depleted patients. Caimmi et al. reported an association between reduced muscle mass and skin involvement in SSc patients [20] while Marighela et al. confirmed that disease severity is a significant risk factor for low muscle mass [21]. In addition, in a previous study, we reported a correlation between low muscularity and DUs [22]. Patients with SSc have been long considered to be at risk for malnutrition, and evidence is accumulating that malnutrition may negatively impact on outcome in SSc patients [23–26] and may be a cause of muscle mass loss [27].

Bearing in mind that low muscularity in SSc patients has a multifactorial pathogenesis, with this study, we wanted to assess whether GIT involvement may be a contributory factor to reduced muscle mass. At the best of our knowledge, this is the first study assessing an association between symptoms related to GIT involvement as assessed by UCLA SCTC GIT 2.0 and reduced skeletal muscle mass as assessed by BIA.

Regarding UCLA SCTC GIT 2.0 assessed gastrointestinal symptoms, in the present study, we found that 13% of patients had severe symptoms, reporting almost the same prevalence (11.3%) observed by Caimmi et al. [20]. Interestingly, we found that UCLA SCTC GIT 2.0 total score and UCLA SCTC GIT 2.0 distention/bloating, although weakly, negatively correlated with FFMI, while only UCLA SCTC GIT 2.0 distention/bloating was significantly higher in SSc patients with reduced FFMI. Of note, although patients with low muscularity have worse disease severity and activity scores (Table 3), in multiple linear regression model, FFMI showed association with UCLA distention/bloating, BMI, and disease duration, but it was not related to disease activity or severity.

The “abdominal distension/bloating” item has been added to 2.0 version in order to better identify patients with symptoms suggestive of three important and inter-related gastrointestinal manifestations of SSc, namely gastrointestinal hypomotility, pseudo-obstruction, and SIBO [3]. Bacterial overgrowth determines malabsorption of fat and vitamins with concomitant production of gas and osmotically active products, which in turn lead to the symptoms of diarrhea, abdominal tenderness, and bloating. Small intestinal bacterial overgrowth is due to delayed gastric emptying and prolonged orofecal transit time and is characterized by an increase in the number and/or an abnormal type of bacteria, which may count up to 60% higher than in healthy controls [28–30]. Dysbiosis-related bloating and distension may induce abdominal pain, in turn causing anorexia and reduced food intake [31], thus favoring muscle loss and possibly explaining the mild association between UCLA SCTC GIT 2.0 distention/bloating and low FFMI observed in the present study. Alternatively, gut dysbiosis in SSc might act similarly to what observed in aging. Recently, the existence of a “gut-muscle axis” has been hypothesized, and changes in gut microbiota have been proposed as potential contributors to age-associated decline in muscle mass and function. In particular, data exist suggesting that in older adults, gut dysbiosis is associated with increased intestinal permeability, favoring the passage of endotoxin and other microbial factors into the circulation, thus prompting systemic inflammation, in turn promoting the loss of muscle mass [32, 33]. Thus, gut dysbiosis might represent a common mechanism for age- and SSc-related loss of muscle. Indeed, some of the changes in gut microbial species observed in elderly people [34, 35] resemble those described in SSc patients [29]. In the present study, the lack of association between FFMI and age would seem to support this hypothesis.

Interestingly, in the present study, no relationship was found between gastrointestinal symptoms and malnutrition, which was diagnosed in up to 23.3% of patients. This observation is in agreement with the available literature. In particular, in the study of Caporali et al. [36], malnutrition was observed in 24 of 160 SSc patients, with a prevalence of 15%. In the multivariate analysis, malnutrition was found independently associated with disease activity. The association between malnutrition and symptoms related to GIT involvement was not statistically significant, although a trend was detected. Consistently with Caporali et al. [36], in our study, UCLA SCTC GIT 2.0 distention/bloating score was higher, although not significantly, in malnourished vs non-malnourished SSc patients, according to both GLIM and ESPEN criteria.

Regarding the discrepancy between malnutrition and reduced FFM, frequently observed in scleroderma patients, our [22] and others’ [20, 21, 37] data highlight that SSc represents a unique study model in which malnutrition and low muscularity are peculiarly embricated and in which onset of muscle loss and malnutrition may not temporarily and causally coincide. Indeed, the mechanisms underlying loss of muscle mass in SSc might be at least partially independent of nutritional factors (e.g., endothelial dysfunctions as microvascular changes and altered angiogenesis involving body organs including skeletal muscle) [22, 37, 38]. The possible role of inflammation in the pathogenesis of muscle loss in SSc patients deserves speculation. Indeed, systemic inflammation is a potent driver of muscle loss in a number of both acute (e.g., ICU) and chronic (e.g., cancer) conditions [7, 39, 40]. In the present study, both the inflammatory indices CRP and ESR were not significantly different between the low and normal FFMI groups. Given the cross-sectional nature of the present investigation, we cannot, in principle, exclude that the inflammatory response might have been higher before the study. However, we believe this is not the case. In fact, as discussed in a previous study of our group [27], the inflammatory response that characterizes SSc is lower than one would expect in an autoimmune disease [41]. This is particularly true in patients with long disease history, as were those included in the present study, whose median disease duration was 10 years. This thinking is further supported by the finding that both CRP and ESR were beyond the upper limit of normality in a minority of the patients included in the present series. A prospective evaluation of the relationships between inflammatory response and body composition in SSc patients would be of undoubted interest, but it was beyond the scopes of the present investigation.

The study has several limitations: first, it is a monocentric study; second, the sample size is small, due to the fact that SSc is a rare disease; third, the fecal microbiota was not assessed, not allowing to confirm the pathogenic mechanisms of distention/bloating.

In conclusion, the present study confirms that low muscularity is a common feature in SSc patients. The pathogenesis of reduced skeletal muscle mass is multifactorial and includes, among others, malnutrition, disease severity and duration, endothelial dysfunction, and GIT. The involvement of the gastrointestinal tract may negatively impact on muscularity through different mechanisms, the individual role of which deserves further investigation.
